# A New Way to Look at Oxidative Stress

**DOI:** 10.1371/journal.pbio.0020374

**Published:** 2004-10-05

Chemical reactions lie at the heart of many biological processes, from photosynthesis and respiration to cell signaling and drug metabolism. Thanks to an atmosphere rich in oxygen, many organisms use oxygen to carry out these life processes. But oxygen metabolism produces highly toxic by-products called reactive oxygen species. When oxidation outpaces detoxifying reactions, oxidative stress occurs, and accumulating reactive oxygen species are free to wreak havoc on cellular machinery.

Cysteine, one of the 20 different amino acids that make up proteins, contains a thiol group, which can be modified upon oxidation. A thiol group can stabilize protein structures by forming covalent disulfide bonds and can mediate cysteine-regulated redox reactions. At the same time, however, the high reactivity of thiol groups makes them also particularly vulnerable to nonspecific reactions during conditions of oxidative stress. Over the past few years, an increasing number of proteins have been discovered that use oxidative thiol chemistry to regulate their protein activity. In *PLoS Biology,* Lars Leichert and Ursula Jakob describe a novel method to monitor thiol modifications in proteins subjected to varying redox conditions in a living organism, the bacteria Escherichia coli. This technique is capable of providing a global snapshot of the redox state of protein cysteines during normal and oxidative stress conditions in the cell.

To detect proteins that have the ability to undergo stress-induced thiol modifications, Leichert and Jakob differentially labeled the thiol groups of thiol-modified and non-thiol-modified proteins. The proteins were then separated on two-dimensional gels based on their charge and molecular weight. If the technique worked, most thiol-modified proteins should be detected in the oxidizing environment of the E. coli periplasm (the region between the cell's membrane layers), and they were.

After proving the method's ability to detect proteins whose thiol groups were oxidized, the next logical step was to determine what proteins DsbA—the enzyme that catalyzes disulfide bond formation in the E. coli periplasm—was targeting. In E. coli mutant strains that lack DsbA, Leichert and Jakob identified a number of proteins with either substantially less or no thiol modification as compared to wild-type (non-mutant) strains, suggesting that these proteins are indeed DsbA substrates.

**Figure:**
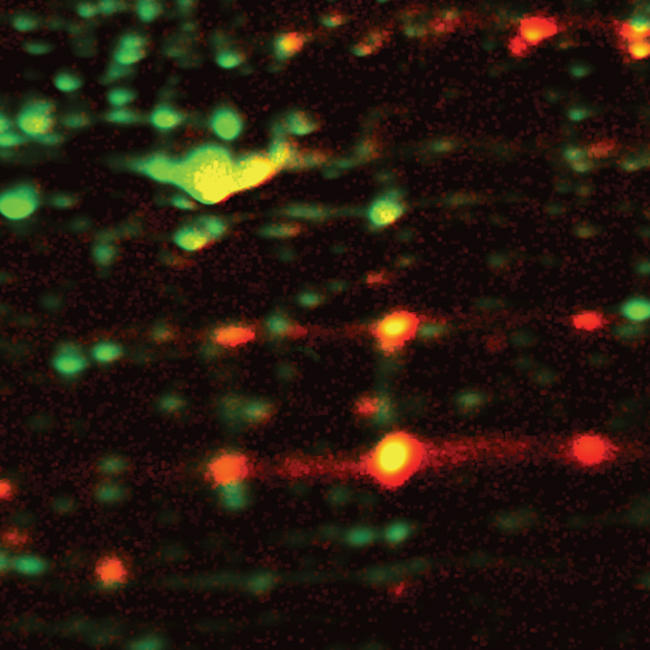
A differential thiol-trapping technique provides a snapshot of the in vivo thiol status of proteins upon variations in the redox homeostasis of cells

In contrast to the periplasm, the E. coli cytoplasm contains several reducing systems. When the researchers tested a mutant strain that lacked the gene for the reducing enzyme thioredoxin, they found that a large number of proteins accumulated in an oxidized state. Many of these proteins have cysteines and require a reduced thiol status for their activity. These results demonstrated that under normal growing conditions, many proteins contain cysteine residues that are vulnerable to even small amounts of reactive oxygen species and so require the constant attention of detoxifying enzymes.

In a final set of experiments, Leichert and Jakob discovered a number of proteins whose thiol groups get specifically modified in the presence of reactive oxygen species. These results start to explain some of the many metabolic changes that occur in oxidatively stressed cells.

Leichert and Jakob's technique should be applicable to many different cell types and organisms and can be used to investigate the in vivo thiol status of cellular proteins exposed to virtually any physiological or pathological condition that is accompanied by oxidative stress. The next step will be to investigate just how thiol modifications mediate the various functions of redox-regulated proteins.

